# Musical ability is associated with enhanced auditory and visual cognitive processing

**DOI:** 10.1186/s12868-015-0200-4

**Published:** 2015-09-16

**Authors:** Caroline Faßhauer, Achim Frese, Stefan Evers

**Affiliations:** Department of Neurology, University of Münster, Münster, Germany; Academy of Manual Medicine, Münster, Germany; Department of Neurology, Krankenhaus Lindenbrunn, Lindenbrunn 1, 31863 Coppenbrügge, Germany

**Keywords:** Music, Musical ability, Event-related potentials, Visual cognition, Auditory cognition

## Abstract

**Background:**

Musical ability has always been linked to enhanced cognitive and intellectual skills. We were interested in the relation between musical ability and short-time cognitive processing as measured by event-related potentials, in particular in visual processing, since previous studies have already suggested such a link for acoustic cognitive processing. We measured auditory and visual event-related potentials as elicited by an oddball paradigm in 20 healthy subjects (10 musicians and 10 non-musicians; 10 female; mean age 24 ± 2 years). In addition, the Seashore test and a test developed by the authors to detect relevant amusia, the latter one with a high ceiling effect, were also applied.

**Results:**

The most important finding was that there is a significant linear correlation between musical ability as measured by these tests and the P3 latencies of both the auditory and visual event-related potentials. Furthermore, musicians showed shorter latencies of the event-related potentials than non-musicians.

**Conclusions:**

We conclude that musical ability as measured by neuropsychological tests is associated with improved short-time cognitive processing both in the auditory and, surprisingly, also in the visual domain.

## Background

Musical ability as a term is used to describe the sensitivity for music, the ability to understand music, and/or the ability to produce music. There is no standard definition, and it is hard to measure musical ability. One can only measure how well a person can perceive musical stimuli such as small changes in pitch, loudness, rhythm, and other sub-domains of music processing. It is generally accepted that some people show higher musical ability than others. But what are the correlates for the term ‘musical ability’ in the human brain? What are the differences between people with and without musical ability?

These questions and many others about music and music processing in the human brain have been a subject of interest in neuroscience research. The methods used in this field range widely from EEG, event-related potentials (ERP), magnetic resonance imaging (MRI) to the different methods of functional imaging. The processing of auditory stimuli and the differences in such processing between musicians and non-musicians have been investigated especially by means of auditory ERP which can objectively quantify latencies of stimulus processing. This evaluation is regarded as a measure for the quality of some aspects of cognitive processing such as stimulus evaluation, cognitive processing speed and internal short-term memory functions.

In general, rare target stimuli and frequent non-target stimuli are presented to subjects in ERP studies. The latencies and amplitudes of the potentials evoked by the target stimuli are analyzed such as the Mismatch Negativity (MMN or N2a) in auditory ERP studies and the P3 in both visual and auditory ERP studies. In previous music research studies, the subjects were either professional and non-professional musicians, people with absolute pitch, or non-musicians.

Looking at the results of different studies [1–5], one can sum up that musicians with absolute pitch ability and musicians with relative pitch seem to have a shorter P3 latency in the auditory evoked ERP and also a smaller P3 amplitude than non-musicians. The P3 could even not be found at all in some musicians with absolute pitch [3] when the method that led to a P3 signal in all musicians with relative pitch was the discrimination between major thirds as non-target and minor thirds as target stimuli. In a timbre discrimination study [6], subjects with absolute pitch showed a shorter P3 latency and a smaller P3 amplitude than other musicians and non-musicians. The subjects had to differentiate between the sound of a tone with the same pitch played on a viola or a cello, on two different kinds of flutes and on two different kinds of tubas. In the last (tuba) part, the musicians showed a significantly shorter P3 latency than the non-musicians.

In a different approach, a piano phrase by Bach was used for eliciting ERP [7]. They presented the original phrase (non-target) or the same phrase with an inharmonic note (target) or the same phrase with an unexpected mordent (target) to a group of musicians and a group of non-musicians. A shorter P3 latency for musicians could not be shown, but instead of that and among other results, a shorter N2 latency was detected. In a study in 1996 [8], the reaction time of musicians was shorter in comparison to non-musicians when the subjects had to discriminate between small differences in frequency. Further, it was incidentally found a significantly decreased P3a latency in musicians (i.e., vocalists and instrumentalists) compared to non-musicians, evoking the P3 by means of pitch deviants [9]. The P3a signal was suggested to be a sensitive index of musical expertise.

All in all, the studies mentioned above suggest a faster discrimination of auditory stimuli in musicians (with or without absolute pitch). None of the studies had analyzed the latencies of visually evoked ERP, and only two [1, 10] have measured the amplitudes of the visually evoked P3 latency in subjects with absolute pitch, which was not found to be changed.

We raised the question whether people with absolute pitch have a different cognitive stimulus processing as measured by ERP, and not just a different processing of auditory stimuli, as most of the studies mentioned above suggest. In an experiment with three participating groups (musicians with absolute pitch, musicians without absolute pitch, and non-musicians), our group was able to show that both people with absolute pitch compared to non-musicians and musicians compared to non-musicians show a significantly decreased P3 latency in the auditory and visually evoked ERP [11]. The P3 amplitudes were not significantly different. In a similar study, our group was able to show a significantly decreased auditory and visually evoked P3 latency in musicians as well as a larger amplitude of the P3 in the auditory domain [12].

In the present study, we were interested in aspects of the specific visual cognitive processing in musicians versus non-musicians. In particular, we aimed to correlate the ERP results of visual and auditory stimulus processing with musical ability as evaluated by a psychometric measure. We chose an oddball paradigm since this is a very easy task not detracting the probands too much. Furthermore, oddball paradigms are often used in research on musical cognitive processing in the past. Since a specific cognitive processing of ERP has been shown for auditory stimuli, a similar result for visual stimuli would suggest that musical ability is associated with a specific cognitive processing in all modalities.

## Results

As shown in Table 1, there were no significant differences between the two groups with respect to age and the scores of the Zerssen scale. All subjects showed a normal mental well-being. This is important since feeling unwell could impair the results of the musical testing.

**Table 1:** Data at baseline of the two subject groups

	Musicians (n = 10)	Non-musicians (n = 10)	Significance
Age	23 ± 2	24 ± 2	ns (p = 0.436)
Sex	5 male/5 female	5 male/5 female	–
Amusia test			
1a. Rhythm	15.5 ± 0.7	9.8 ± 1.8	p < 0.001
1b. Metrum	16.0 ± 0.0	11.4 ± 2.9	p < 0.001
2. Comparison of melodies	15.5 ± 0.7	12.6 ± 1.8	p < 0.001
3. Emotion	11.2 ± 0.9	11.0 ± 0.9	ns (p = 0.684)
4. Pitch	12.0 ± 0.0	10.5 ± 1.3	p = 0.007
5. Identification of melodies	13.3 ± 0.7	11.9 ± 1.6	ns (p = 0.052)
Total score	83.5 ± 1.2	67.2 ± 5.7	p < 0.001
Seashore test			
1. Pitch	46.1 ± 2.9	34.6 ± 8.6	p = 0.001
2. Loudness	45.4 ± 1.8	42.4 ± 3.0	p = 0.011
3. Rhythm	28.6 ± 1.0	25.7 ± 3.0	p = 0.005
4. Duration of a tone	45.0 ± 2.8	42.2 ± 2.3	p = 0.029
5. Timbre	42.3 ± 3.1	37.1 ± 6.0	p = 0.043
6. Tonal memory	26.0 ± 2.3	15.9 ± 6.7	p = 0.001
Total score	233.4 ± 6.6	197.9 ± 22.7	p < 0.001
Zerssen 1	29.9 ± 5.6	32.8 ± 6.8	ns (p = 0.315)
Zerssen 2	31.3 ± 9.9	32.2 ± 6.0	ns (p = 0.393)

Comparison between groups by Mann–Whitney U test

### Musical ability

The scores in the self-developed amusia tests and in the Seashore test are shown in Table 1. In average, the group of musicians scored 83.5 ± 1.2 out of 86 points in the amusia test as opposed to the group of non-musicians with a score of 67.2 ± 5.7 (p < 0.001). In the Seashore test, the results were significantly different between both groups for each of the six categories. The musicians’ total score was 233.4 ± 6.6, the non-musicians’ total score was 197.9 ± 22.7 (p < 0.001).

### Visually and auditory evoked ERP

Table 2 shows the data of the visual ERP (elicited by an oddball paradigm with red light as target), Table 3 shows the data of the auditory ERP (elicited by an oddball paradigm with a high tone as target), presented separately for both groups. The analysis of the visual ERP resulted in significant differences in the P3 latency and the P3 habituation (i.e., difference of P3 latency in two different trials) after the target stimuli: the P3 latency was 390 ± 33 ms in the musician group and 411 ± 22 ms in the non-musician group (p = 0.043). The P3 latency habituation was 0.6 ± 7.8 ms (musicians) and 7.3 ± 11.0 ms (non-musicians) with a significance of p = 0.018. The evaluation of the mean choice reaction time, the P3 amplitude, and the signals occurring after the frequent stimulus did not result in any significant differences.

**Table 2:** Data of visually evoked event-related potentials (oddball paradigm) recording presented separately for both subjects group

	Musicians (n = 10)	Non-musicians (n = 10)	Significance
Frequent stimulus			
P1 latency (ms)	122 ± 11	81 ± 0	ns (p = 0.500)
N1 latency (ms)	165 ± 23	169 ± 24	ns (p = 0.604)
P2 latency (ms)	252 ± 14	251 ± 16	ns (p = 0.837)
N2 latency (ms)	305 ± 19	301 ± 18	ns (p = 0.755)
P3 latency (ms)	394 ± 23	383 ± 52	ns (p = 0.902)
Infrequent stimulus			
P1 latency (ms)	94 ± 27	116 ± 0	ns (p = 0.667)
N1 latency (ms)	156 ± 23	168 ± 20	ns (p = 0.243)
P2 latency (ms)	228 ± 9	239 ± 15	ns (p = 0.105)
N2 latency (ms)	272 ± 16	277 ± 15	ns (p = 0.631)
P3 latency (ms)	390 ± 33	411 ± 22	p = 0.043
P3 amplitude (µV)	14 ± 6	12 ± 4	ns (p = 0.253)
P3 habituation (ms)	0.6 ± 7.8	7.3 ± 11.0	p = 0.018
Mean choice reaction time (ms)	381 ± 45	405 ± 58	ns (p = 0.436)

**Table 3:** Data of auditory evoked event-related potentials (oddball paradigm) recording presented separately for both subjects group

	Musicians (n = 10)	Non-musicians (n = 10)	Significance
Frequent stimulus			
P1 latency (ms)	60 ± 2	63 ± 3	ns (p = 0.229)
N1 latency (ms)	125 ± 22	112 ± 6	ns (p = 0.247)
P2 latency (ms)	229 ± 27	199 ± 17	p = 0.021
N2 latency (ms)	302 ± 11	259 ± 43	ns (p = 0.190)
P3 latency (ms)	360 ± 29	332 ± 48	ns (p = 0.400)
Infrequent stimulus			
P1 latency (ms)	54 ± 5	71 ± 29	ns (p = 0.413)
N1 latency (ms)	109 ± 10	109 ± 9	ns (p = 0.631)
P2 latency (ms)	171 ± 20	180 ± 13	ns (p = 0.165)
N2 latency (ms)	218 ± 22	230 ± 15	ns (p = 0.123)
P3 latency (ms)	328 ± 34	360 ± 10	p = 0.019
P3 amplitude (µV)	13 ± 5	12 ± 4	ns (p = 0.971)
P3 habituation (ms)	−5.8 ± 19.6	17.4 ± 11.2	p = 0.009
Mean choice reaction time (ms)	345 ± 43	349 ± 56	ns (p = 1.000)

The latencies in the auditory ERP showed similar differences between the two groups: the P3 latency in the musician group was 328 ± 34 ms as opposed to 360 ± 10 ms in the non-musician group (p = 0.019). The P3 latency habituation differed by 23.2 ms between the two groups with a P3 habituation of −5.8 ± 19.6 ms in the group of musicians (p = 0.009).

### Correlation of P3 latency and musical ability

**Fig. 1:**
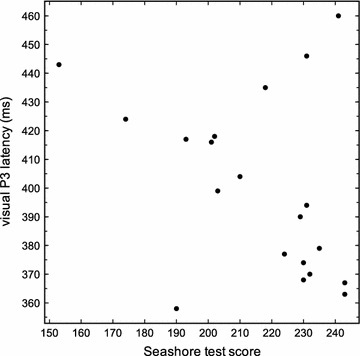
Correlation between P3 latency of the visual event-related potentials and the total score of the Seashore-test (r = −0.470 and p = 0.036; Spearman-rank-coefficient)

**Fig. 2:**
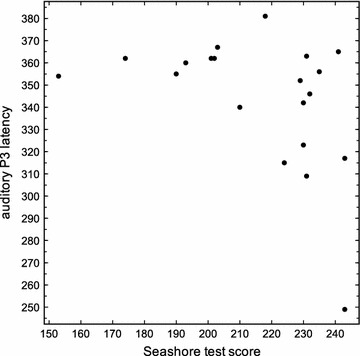
Correlation between P3 latency of auditory evoked event-related potentials and results in the Seashore-test (r = −0.434; p = 0.038; Spearman-rank-coefficient)

We further calculated the correlation between the results in the Seashore test and the P3 latencies. As shown in Figs. 1 and 2, there was a negative correlation between the P3 latency of the visual ERP and the total score of the Seashore test (r = −0.470 and p = 0.036; Spearman-rank-coefficient), as well as between the P3 latency of the auditory ERP and the total score of the Seashore-test (r = −0.434 and p = 0.038). This means that a higher musical ability is correlated to a shorter visual and auditory P3 latency.

## Discussion

### Testing musical ability

In the amusia test, the musicians scored significantly higher than the non-musicians, except for the categories ‘emotion’ and ‘identification of melodies’. The purpose of this amusia test was to rule out a clinically relevant amusia in the non-musician group. The average score of 67 is within the normal range of >60 [13]. Therefore, the non-musician group did not have a relevant impairment of musical ability. One has to take into account that this test has a high ceiling effect and was not designed for measuring small interindividual differences in musical ability, although different categories of musical ability are tested. This explains why in two of the categories the results were not significantly different between musicians and non-musicians; and this is also the reason why we used the Seashore test results for further analysis of musical ability and the correlation analysis with the P3 latencies.

The musicians scored significantly higher in the Seashore-test. This confirms that the group of musicians has a higher musical ability than the group of non-musicians. But what are the reasons for this? Several studies have investigated the question whether a higher degree of musical ability in musicians could be a result of practice-induced cortical plasticity or an innately given talent [14–18].

In two studies [16, 17], non-musicians who had learned to play a certain rhythmical sequence or different melodic sequences showed an increased MMN in response to rhythm or pitch incongruities. Another group of non-musicians, who had just listened to the other group practising, did not show an increased MMN response. The authors interpret this finding as a sign of practice-induced cortical plasticity. Therefore, we assume that the musicians’ higher score in the Seashore test is a result of the musical training of 14 ± 2 years on average, although parts of the musical ability could also be a genetically driven talent.

It was shown that professional musicians compared to amateur and non-musicians have an increase in grey matter volume in several brain regions such as motor, visual-spatial, and especially auditory regions [15]. Because of the associations found between structural differences, musician status, and practice intensity, the authors interpret their results as practice-induced adaptations of the brain. In 2009, changes induced by private keyboard lessons in young children who had no prior musical training were studied [18]: the results showed that instrumental children (versus children in a control group) showed a greater size of certain motor and auditory areas after 15 months of training and that their outcomes in motor and melody/rhythm tests also improved significantly. Again, this supports the hypothesis of practice-induced musical skills going along with structural brain changes.

### P3 latency and musical ability

The P3 response to the target stimuli of both the visual and auditory ERP was significantly earlier in the group of musicians. This result confirms the results of previous studies [11, 12]. An early P3 response indicates fast stimulus processing and discrimination. Thus, musicians seem to be able to discriminate faster between auditory stimuli. Considering their long and special exposition to auditory stimuli in form of practising and listening to music, this result does not seem surprising.

Even though the mechanisms that determine latency of the P3 response have not yet been fully understood, there are several studies which have shown differences in the (auditory) brain structures of musicians. Several studies suggest a practice-induced brain plasticity and thus a functional and structural difference in auditory brain areas of musicians versus non-musicians [14–17, 19]. The fact that musicians show an earlier P3 latency than non-musicians and thus have a faster auditory stimulus processing may be associated with those results. Supporting this notion, a negative correlation between P3 latency and the years of musical training was found [12].

In our study, not only the auditory P3 latency was earlier in musicians but also the visual P3 latency. Musical training and/or musical ability therefore seem to affect also the visual perceptive system. This also goes along with the results of other studies concerning the musicians’ visual abilities. It was shown that musicians outperformed non-musicians in the ability of perceptual speed, which requires quick visual information processing [20]. Visual memory was also reported to be better in musicians than in non-musicians [21–24]; even primary visual perception [25] and somatosensory cognitive processing [26] were reported to be enhanced in musicians. However, these studies used psychometric measures and did not evaluate cognitive processing in an objective way. A difference in grey matter volume in visual-spatial areas was found [15], going along with other results [27] indicating an improvement of visual-spatial tasks in musically trained children.

### Correlation of Seashore test results and P3 latency

In our study, we were also able to link the early P3 responses in the auditory and visual paradigms to the results of the Seashore-test and found a negative correlation between the data. In studies on musicians, a musician is usually defined as a subject who has learned to play an instrument for a certain amount of years and musicians can be classified as ‘professional’ or ‘amateur’. These definitions usually are the criteria for the participation of musicians in studies, but having had music lessons for a certain amount of years does not necessarily yield in a high musical ability. With our results, we showed that not just the status ‘musicianship’, but the amount of musical ability as measured by a psychometric test is related to the improved cognitive processing of musicians versus non-musicians.

All in all, people with high musical ability seem to have a faster auditory and visual cognitive processing. The question is, however, whether this connection between musical ability and cognitive processing is inherited or whether the acquisition of musical ability (i.e. long-term practice of an instrument) leads to an enhancement of cognitive processing. At this time, one can only speculate about the answer: on the one hand, fast cognitive processing is probably helpful for learning an instrument, which requires fast interaction of auditory, motor, and visual stimuli. One could even conjecture that musical ability is a congenital ability of cognitive processing. Some studies have shown evidence that the conditions upon which musical ability develops are innately predetermined. Hassler for example showed that a certain androgynous level of testosterone is characteristic of musicians, and that differences between musicians and non-musicians are possibly formed prenatally under the influence of hormones [28].

On the other hand it also seems plausible that by practicing an instrument the processing of all these stimuli becomes faster, which then might lead to the improvement of auditory and visual abilities, as shown in the studies mentioned above. In an MEG study, an enhanced auditory evoked magnetic field response concerning violin tones was shown in children who had been musically trained for 1 year [29]. There has also been a study not connected to music, which showed alterations in adults’ ERP in response to an auditory discrimination task [30]. Hence, it has been shown before that training can have an influence on stimulus processing and therefore it is plausible that musical training which leads to a higher amount of musical ability could have an effect on auditive and visual processing [31, 32].

All in all we suppose that there might be certain inherited conditions such as fast cognitive processing upon which musical ability develops but that a stimulating environment as well as long-term musical practice also leads to an improvement of cognitive processing and/or aptitude. This is also important for disturbances of cognition e.g. after stroke, after brain injury, or in different types of amusia [33, 34].

### P3 latency habituation

In our study, we calculated the P3 latency habituation and found that musicians showed a habituation for both the visual and the auditory latencies close to zero, whereas in non-musicians it was significantly higher. There is evidence that, in normal subjects, the visual and auditory P3 latency increases over trial blocks [35–38]. A loss of habituation in the visual P3 latency of migraine patients during the migraine interval was shown [37], similar to our findings of a loss of habituation in musicians. We believe that the loss of habituation and the generally decreased P3 latency in musicians indicate that musicians have a remarkable cognitive processing with the ability of keeping the processing of stimuli faster for a longer time than normal subjects. This could be an indicator of an improved working memory in musicians, as it has been reported by some authors [12, 39, 40]. Keeping the results from migraine patients in mind, it would be interesting to study whether in musicians a similar effect like the normalization of the habituation during a migraine attack could be observed after an interval of increased alertness.

A loss of cognitive habituation (or higher cognitive excitability) also means that stimuli on different levels might be processed faster and more accurate for a certain amount of time. This can be regarded as an advantage for musicians, e.g. when playing in an orchestra or playing both complex rhythmic and complex melodic music.

### Limitations

Our study has some limitations which have to be considered when interpreting our results. First of all, the concept of musical ability is still under discussion. There is no clear definition and no commonly accepted measure to evaluate musical ability. The sub-domains of musical ability as measured in both the Seashore test and the amusia test only include some aspects of musical ability which are commonly accepted as basic skills of musical ability (e.g., rhythm, loudness, pitch, duration of a tone, tonal memory); other aspects such as creativity, emotional perception of music, the ability of interpreting music etc. are not included because these aspects are hard to measure in an objective way. Nonetheless, they determine the amount of one’s musical ability. We accept that our study did not comprise all aspects of musical ability.

A second problem is the procedure of testing musical ability. The test results depend strongly on the cooperation and motivation of the subjects, which in turn is hard to evaluate. This leads to a reduced objectivity of the testing of musical ability.

Third, when looking at the P3 latency results one has to consider that they only apply to our testing range. Since we looked at P3 latencies that were within in the normal range and only included healthy subjects one cannot transfer the results of the correlation analysis to subjects with pathological P3 latencies, i.e. pathological P3 latencies certainly do not yield in a total lack of musical ability.

Fourth, it is not clear whether or to which extent a decreased P3 latency has an impact on everyday life. A decreased P3 latency implies a faster cognitive processing. Some authors suggest an improved working memory, but there is still an ongoing discussion about this (e.g., [12]).

## Conclusion

Our most important finding is that measures in tests of musical ability are associated with decreased latencies in auditory and visual ERP suggesting that musical ability is associated with a general enhancement of cognitive processing.

Regarding the auditory P3 latency, we were able to reproduce findings of previous studies [1–5]. Additionally, we were able to show a significant correlation between the level of musical ability as measured by the Seashore test and both the visual and the auditory P3 latency. Also, a significant difference in habituation of the visual and auditory P3 latencies between non-musicians and musicians could be observed. These results cannot be attributed to a different mental state of the two groups, as this would have lead to significantly different results between the two groups in the Zerssen self-rating Scale.

## Methods

### Participants

We compared a group of musicians (N = 10; 5 male) to a group of non-musicians (N = 10; 5 male). The mean age of the two groups was 23 ± 2 and 24 ± 2 years, respectively. Musicians were defined by having received at least 10 years of private music lessons (mean 14 ± 2 years) and being active musicians (i.e., members of orchestras, chamber music groups, or choirs) at the time of the study. The non-musician group had no or very few (less than 2 years) music lessons in their lives. Exclusion criteria for the participation in the study were psychiatric or neurological disorders including migraine and epilepsy and medication on a regular basis except for oral contraceptives. Consumption of alcohol, nicotine, or caffeine was not allowed on the day of the experiment.

The experiment was always performed in the afternoon in a room without daylight. The room temperature was always the same. For the last part of the experiment, the recording of the ERP, the lights were turned off. All participants gave written informed consent. The study was approved by the local ethics committee.

### Psychometric testing

In the beginning, the participants had to complete the Zerssen self-rating scale [41]. The Zerssen scale measures the actual mental well-being with a score between 0 and 56. In the validation sample, a mean score of 12 ± 10 was described for healthy subjects. Only subjects with a score within the normal mean plus/minus one standard deviation in both measures were included in the study. Feeling mentally not well would impair the results of neuropsychological testing in an unappropriate way.

Then, two tests of musical ability were conducted. The first one was a test on amusia developed by the authors [13]. In this test, the participants had to perform different tasks in the following five different categories: (1) production of rhythm/metrum by repeating different rhythms (knocking with a pin; (2) comparison of melodies (listening to two short melodies and deciding whether they are different or not); (3)emotional impression of music (listening to short pieces of new music which had to be categorized as happy, angry, frightening etc.; the impression could be said to the examiner or shown by different pictures showing the emotion); (4) pitch (comparison of two tones and deciding whether they have the same pitch or a different pitch); (5) identification of 14 commonly known melodies such as “Frère Jaques” or “Yesterday”. The maximum score of this test is 86 points, the higher the score the better the musical ability. This test was originally designed to detect amusia in neurological patients. It has a high ceiling effect, which means that a score of less than 90 % of the maximum denotes a relevant impairment. Therefore, the test is or poor value to detect small differences between individuals.

In order to evaluate the subjects’ musical ability in a previously designed test, we used the “Seashore test for Musical Ability” [42] as a second test. It contains six different categories: (1) pitch; (2) loudness; (3) rhythm; (4) duration of a tone; (5) timbre; (6) tonal memory. The maximum score is 260. The subjects have to differentiate between small changes in pitch, loudness, rhythm etc. In the category pitch, for example, 50 pairs of tones are presented, differing in frequency from/between 17 and 2 Hz. The subjects are asked whether the second tone is lower or higher in frequency than the first. This test shows a nearly parametric distribution of scores and has no defined scores for amusia.

### ERP recording

The second part of the experiment was the recording of visually and auditory evoked ERP. The subjects had to sit in a comfortable chair in a dark room. For the visually evoked ERP the subjects were looking at a 30 cm × 30 cm video screen, standing approximately 150 cm in front of them. Two trains of 200 flashes of light each with a 3 min break in between the trains were presented in a random order. We used 15 % red (target) and 85 % white (non-target) flashes of light. The duration of a single flash was 100 ms and the interval between two flashes was 1800 ms. Subjects were asked to press a button with their dominant hand whenever they observed a red flash. For the auditory evoked ERP, two trains of 200 tones each (3 min break between the two trains) were presented to the subjects. The target tones (15 %) had a pitch of 600 Hz, the non-target tones had a pitch of 325 Hz, they were also randomly mixed. Again, the subjects had to press a button whenever a target stimulus occurred. The duration of a tone was 100 ms, and the interval between two stimuli was 1800 ms.

In both ERP measurements (auditory and visual), the EEG was recorded by an amplifier using Ag/AgCl surface electrodes, which were placed at centroparietal (Pz), centrocentral (Cz), and frontocentral (Fz) (=recording electrodes) according to the international 10–20 system. They were linked to the mastoid (=reference electrode). EOG was also registered in order to exclude EEG periods with eye movement artifacts from the ensuing averaging process. A high-frequency filter was set at 70 Hz and a low-frequency filter at 0.1 Hz. The EEG was stored digitally. EEG periods of 300 ms before and 1100 ms after onset of the stimulus were averaged separately for the target and non-target stimuli.

The evaluation of the ERP latencies was performed by a physician who did not conduct the experiment and who did not know which subjects were musicians and which ones were not. The components of the ERP following the red/high and white/low stimuli were evaluated. We determined the latencies of the P1, N1, P2, N2, and P3 components and the amplitude of the P3 component, further we measured the mean choice reaction time (i.e., the time between onset of the target stimulus and pressing of the button) according to international recommendations [19]. For both the visual and the auditory ERP, the curves of the first and of the second train (200 stimuli), separated in target and non-target stimuli, were averaged and then the latencies, the P3 amplitude and the mean choice reaction time were measured. By calculating the difference of P3 latency between the first and the second train, the P3 habituation could also be evaluated. The P3 component of the ERP was chosen as the main parameter since it is associated with endogenous (mainly stimulus independent) cognitive processing whereas the P2 and the N2 components are exogenous (i.e., mainly dependent from the physical properties of the stimulus).

### Statistical analysis

We used non-parametric tests to analyze the correlation between the different test scores and the ERP parameters and to analyze group differences between musicians and non-musicians. For correlation analysis, the Spearman rank coefficient was calculated. For group comparisons, we used the Mann–Whitney U test. Multiple comparisons were corrected by Bonferroni correction. All calculations were performed by the program SPSS version 18.0. Significance level was set at p = 0.05.
